# Lemur tyrosine kinase-3 (LMTK3) in cancer and evolution

**DOI:** 10.18632/oncotarget.291

**Published:** 2011-06-16

**Authors:** Justin Stebbing, Aleksandra Filipović, Georgios Giamas

**Affiliations:** ^1^ Department of Oncology and Surgery, Imperial College, Hammersmith Campus, London

Estrogen receptor positive breast cancer remains the commonest form of metastatic incurable disease. Although estrogenic signalling in breast cancer is one of the most critical oncogenic pathways known, there are no specific therapies designed to overcome endocrine resistance. This is in contrast to the situation in less common types of breast cancer including triple negative disease (PARP inhibitors) and HER2 positive relapsed tumours (T-DM1 and neratinib, amongst others).

As kinases are some of the most common drug targets [1], we sought to identify previously uncharacterized molecules that regulate the estrogen receptor-α (ERα) [2,3]. To start with, we performed a ‘high risk’ screen using expression of the estrogen responsive gene pS2 as a read-out, as opposed to standard sets of results such as measures of cell proliferation. Known kinases including MAPK3 and Akt that modulate activity of ERα via phosphorylation were used as controls.

Changes in DNA sequences have been proposed to play distinctive roles in adaptive evolution. While presumably all proteins must have been positively selected for their biochemical functions at some time in the past, only a very few show protein-coding sequence evidence of such adaptive evolution; we thought it notable that LMTK3 fell into this category. An excess of nonsynonymous (coding) over synonymous (non-coding, silent) codon mutations was used to identify sites of positive selection and clarified that only LMTK3 amongst all the molecules we studied had adaptively evolved. We therefore decided to focus our attention on LMTK3; in doing so, we attempted to explain in part the unique susceptibility of humans to ERα positive breast cancers.

Interestingly, reproductive proteins are among the fastest evolving in the genome and several models have been proposed to explain this including sexual conflict and sexual selection [4]. It is therefore of enormous interest that an ERα modulating kinase was the only protein we observed to be positively selected in our screen. It is also significant that the LMTK1 and LMTK2 isoforms have not been positively selected between human and chimpanzees; they are well-conserved. We demonstrated that positive selection occurred across the LMTK3 gene except for the catalytic core domain, presumably altering substrate binding characteristics of human vs. chimpanzee LMTK3. While the selective pressure that drove this adaptive event is at present unclear, an evolutionary trade-off may have led to increased human susceptibility to this disease.

Having shown that methylation was unlikely to play a role here but the presence of certain single nucleotide polymorphisms (SNPs) may be relevant, we compared human and non-human primate (NHP) SNPs. Most humans we examined had the ‘protective’ TT allele, while the less-susceptible NHPs lack the protective TT allele, perhaps as a result of selective pressure to counter possible deleterious effects of sequence changes to human LMTK3. In effect we are postulating two selective pressures/events: one on the protein sequence/structure and another on the frequency of the TT SNP in the LMTK3 intron. The second event was thus likely compensatory for changes that occurred in the first event, which altered the protein itself. We acknowledge that it is of course possible that the positively selected amino acid replacements in human LMTK3 are not responsible for the documented human susceptibility to breast cancer; it is still the case however, that the TT allele our data suggests to be protective in humans is present at far lower frequencies in the less-susceptible NHPs, suggesting that the difference in allelic frequency compensates at least in part for greater human susceptibility to breast cancer. The allelic differences in LMTK3 intron are we believe likely to impact gene expression/regulation of LMTK3 or nearby genes.

In aggregate, these data demonstrate the use of evolutionary analyses to refine results and select proteins for further study. Such data will also assist in discovery of relevant protein-protein interacting regions. We think our subsequent mechanistic and clinical outcomes data attest to this, though there is much to be learnt to clarify mechanisms of endocrine resistance. The identity of a new kinase with a role in this process may be fundamental to future work in this area.
